# Evaluating the Impact of Flexible Alcohol Trading Hours on Violence: An Interrupted Time Series Analysis

**DOI:** 10.1371/journal.pone.0055581

**Published:** 2013-02-15

**Authors:** David K. Humphreys, Manuel P. Eisner, Douglas J. Wiebe

**Affiliations:** 1 Institute of Public Health, University of Cambridge, Cambridge, United Kingdom; 2 Institute of Criminology, University of Cambridge, Cambridge, United Kingdom; 3 Department of Biostatistics & Epidemiology, University of Pennsylvania, Philadelphia, United States of America; California Pacific Medicial Center Research Institute, United States of America

## Abstract

**Background:**

On November 24^th^ 2005, the Government of England and Wales removed regulatory restrictions on the times at which licensed premises could sell alcohol. This study tests availability theory by treating the implementation of Licensing Act (2003) as a natural experiment in alcohol policy.

**Methods:**

An interrupted time series design was employed to estimate the Act’s immediate and delayed impact on violence in the City of Manchester (Population 464,200). We collected police recorded rates of violence, robbery, and total crime between the 1st of February 2004 and the 31st of December 2007. Events were aggregated by week, yielding a total of 204 observations (95 pre-, and 109 post-intervention). Secondary analysis examined changes in daily patterns of violence. Pre- and post-intervention events were separated into four three-hour segments 18∶00–20∶59, 21∶00–23.59, 00∶00–02∶59, 03∶00–05∶59.

**Results:**

Analysis found no evidence that the Licensing Act (2003) affected the overall volume of violence. However, analyses of night-time violence found a gradual and permanent shift of weekend violence into later parts of the night. The results estimated an initial increase of 27.5% between 03∶00 to 06∶00 (ω = 0.2433, 95% CI = 0.06, 0.42), which increased to 36% by the end of the study period (δ = −0.897, 95% CI = −1.02, −0.77).

**Conclusions:**

This study found no evidence that a national policy increasing the physical availability of alcohol affected the overall volume of violence. There was, however, evidence suggesting that the policy may be associated with changes to patterns of violence in the early morning (3 a.m. to 6 a.m.).

## Introduction

Violence and aggressive behaviour has a well established association with alcohol consumption [1], [2], [3]. This relationship has been shown to be causal in some laboratory settings [4], but in the natural environment, alcohol-related violence is thought to be mediated by a complex web of personality, cultural and situational factors [5], [6]. Throughout history, societies have developed regulatory controls that restrict the times and places at which alcohol can be sold, in an attempt to reduce social disorder [7]. These measures are consistent with ‘availability theory’, proposing that the greater availability of alcohol in the population, the greater the prevalence of problems stemming from alcohol consumption [8]. In recent years, many western governments have relaxed restrictions on the physical availability of alcohol [9] and, in some cases, implemented policies that increase public access to alcohol [10]. Sudden changes to regulations governing when and where alcohol can be sold can provide a unique opportunity to study the relationship between alcohol availability and violence [11].

At the turn of the millennium, the growing prevalence of alcohol related problems led to increased public and political concern in England and Wales. A Government report estimated the annual cost of alcohol related harm to exceed twenty billion pounds, with 60% (£12 billion) being attributed to the costs of alcohol-related crime and disorder [12]. At around the same time, a Home Office White Paper (‘Time for Reform’) was published recommending radical changes to regulatory framework governing alcohol availability [13]. In 2003 these proposals were accepted and formalised in The Licensing Act (2003) (hereafter ‘the Act’), which was designed to release the leisure trade from unnecessary bureaucracy, to instil a safer drinking culture, and to reduce crime and disorder [14].

Contrary to traditional restrictive controls, the Act proposed to reduce crime by removing restraints, rather than by adding them. The rationale used to justify these changes evolved from three reports observing overcrowding and other late-night bottlenecks (e.g. at food outlets and taxi ranks) at fixed closing times (i.e. 23∶00 and 02∶00), which were believed to fuel violence and disorder [15], [16], [17]. These reports suggested that removing fixed closing times would help to stagger crowd dispersal from licensed premises, reducing violent behaviour as a consequence [14]. In November 2005 the Act was implemented, removing restrictions on trading hours for alcohol outlets, thus potentially increasing the physical availability of alcohol. Under the principles of availability theory, a significant increase in the availability of alcohol should lead to increased rates of physical and social harm. This study uses the Act as an opportunity to evaluate a government policy that proposed to reduce violence by increasing the availability of alcohol. To do this we examined whether and how trends in violence changed in a large city in northern England after implementation of the Act.

### Effects of Increased On-premise Trading Hours on Violent Crime: Previous Research

Several reviews have summarised the impact of changes to on-premise trading hours on alcohol-related harm [10], [18], [19], [20]. Overall, empirical evidence supporting the availability hypothesis seems particularly strong for road traffic harm and excessive alcohol consumption [19]. However, fewer studies have evaluated the impact of extended trading hours on violent behaviour.

A recent study found only six studies evaluating the impact of trading hour extensions on violent behaviour, some of which offer support for the availability hypothesis [21]. In the same study, researchers predicted a 17% increase in violent assault for every one hour of extended opening in a sample of Norwegian cities [21]. A study conducted in Australia found a statistically significant increase in violent assault around premises with extended trading permits [22], and a 31% increase in emergency room attendances on weekends was observed in Iceland when restrictions on closing hours were removed [23]. Alternatively, however, a British study that examined the impact of a one hour extension in trading times found no significant impact on reported violence [24]. On the whole, the evidence from current reviews offers some support for the hypothesis that increased hours of sale result in greater violent harm, however the overall picture lacks consistency [21].

The review also included studies examining the impact of the Licensing Act (2003) on violence [21]. However, their synthesis included only three of the ten evaluations available to date. To examine the results of the evidence in its entirety, we have summarised all studies examining the impact of on-premise availability extensions (see Table S1). Four studies found no significant change to patterns of violence following the implementation of the Act [25], [26], [27], [28]. In line with availability theory, three studies reported statistically significant increases in violence [29], [30], [31]. Conversely, three studies found reductions in violence, contradicting availability theory (whilst offering partial support for the Government rationale) [32], [33], [34]. In addition, several studies found empirical evidence of changes to the hourly distribution of violence in the post-intervention period. Consistent with findings from international literature [20], [22], these studies show patterns of violence spreading later into the evening [26], [27], [31], [33].

To date, there are no randomised controlled trials of changes to licensed trading times, therefore the evidence base relies heavily on opportunistic studies from several natural experiments around the world. The lack of consistency within the literature may be attributed to various methodological problems inherent within complex alcohol policy evaluations [14], [18], [21]. These studies tend to employ weak methodological designs that lack suitable control conditions. Even in cases where comparison conditions have been incorporated, the proximity of treatment and control areas make contamination a problem. Furthermore, many studies are retrospective and therefore unable to conduct process evaluations detailing the implementation of new measures. Until now, only two studies have employed more robust non-randomised designs such as interrupted time series modelling, neither of which were applied to the Licensing Act [21], [22]. In the absence of prospectively designed randomised control studies, more robust nonrandomised studies are required to help generate evidence.

## Materials and Methods

### Design

We treated the removal of trading hour restrictions that resulted from the Act as a natural experiment in order to test two competing hypotheses: first, that flexible trading hours would lead to a reduction in levels of violence (as predicted by the Labour Government [13]); and secondly, that flexible trading hours would increase levels of violence (as predicted by availability theory [8]). Each hypothesis was tested using an interrupted time series (ITS) design, which has been recommended as a suitable evaluation design in cases where it is difficult to find appropriate control conditions [11], [35], [36], [37], [38]. Using a period of time as the unit of analysis, ITS designs use multiple measures of a pre- and post-intervention outcome variable to estimate intervention effects.

We obtained recorded crime incident data from Greater Manchester Police between 1^st^ of February 2004 to the 31^st^ of December 2007. In order to generate measures comparable to other evaluations [25], [27], [28], [39], we recoded crime into categories defined by the 2008 Home Office counting rules [40]. The data included detailed information on the date and time of the incident, which made it possible to aggregate ‘violence against the person’ to weekly units; this was used as the primary dependent variable. The long study period enabled us to generate a series of 204 weekly time points (95 pre- and 109 post-intervention), which exceed minimum recommended sample size (i.e 50 observational units) for ARIMA impacts assessments [36], [41], [42]. We separated our analyses by weekday (Sunday 12∶00 p.m. to Friday 11∶59 a.m.) and weekends (Friday 12∶00 p.m. to Sunday 11∶59 a.m.) due to well known differences in routine patterns of public alcohol consumption [43], [44]. Furthermore, in order to examine changes to the temporal distribution of violence we performed separate analyses on individual time segments. We separatedq night-time hours into four segments: 18∶00–20∶59, 21∶00–23.59, 00∶00–02∶59, 03∶00–05∶59.

A common limitation of natural experimental studies is the failure to construct plausible counterfactual conditions, thus making it difficult to take account of historical confounding factors [45]. This is a problem common to studies examining policy interventions which are implemented simultaneously across a population [35]. A partial solution to this problem is to include non-equivalent dependent variables [35], [46], [47]–variables not expected to respond to the intervention, but exposed to the same historical validity threats [45]. In this study we used two non-equivalent variables to control for confounding factors, these were: robbery, and total crime. Although it is possible that many types of criminal behaviour could be associated with alcohol consumption, studies of drug use in English and Welsh arrestees calculated attribution fractions for alcohol in robbery (13%) and total crime (22%), which were far lower than for violence (37%) [48]. To our knowledge there is no evidence that robbery or total crime rates are associated with changes in closing hours to the same extent as violent assault. Variations in these types of crime are therefore less likely to change as a result of the Act, but are potentially responsive to cyclical factors that may confound the Act’s impact on violence. The annual frequencies for these variables are presented in Table 1.

**Table 1:** Crime frequencies (2004–2007).

	2004		2005		2006		2007		Total	
	N	%	N	%	N	%	N	%	N	%
*Violence*	12744	15.2	13611	15.2	14823	15.9	13338	16	54516	15.6
*Robbery*	3425	4.1	3505	3.9	3801	4.1	3365	4	14096	4
*Total crime*	83692	100	89461	100	93106	100	83316	100	349575	100

In an attempt to identify any confounding events occurring during the study period that might serve as plausible alternative explanations for our findings [35], [49] (i.e. historical threats to validity), we researched local events that occurred between 2004 and 2008 and found two such factors. The first was the Smoking Ban of 2007, which prohibited smoking within licensed premises, causing individuals to crowd in streets. The second was a series of short-term Alcohol Misuse Enforcement Campaigns (AMECs), during which extra police and local authority enforcement resources targeted alcohol related disorder. Each was included within our analytical structure.

### Statistical Analysis

We tested our hypothesis using interrupted autoregressive integrated moving average (ARIMA) models [37], [38], [50]. For each time series, conventional methods were used to identify the nature of the time series. Logarithmic transformations and differencing was applied when necessary to achieve time series that were normally distributed and stationary in level and variance. Autocorrelation functions (ACF) and partial autocorrelation functions (PACF) of the time series and of the residuals from each ARIMA model were used to identify evidence of seasonality and to test model fit.

Transfer functions were then incorporated into the ARIMA models to perform the impact assessment component of the analysis. Each time series was modelled using three types of transfer functions to test for evidence that the date of the licensing act was associated with either an abrupt permanent, a gradual permanent, or an abrupt but temporary impact on violence (or other crime) [37], [38]. We used this strategy because a process evaluation (reported elsewhere, [14]) found considerable variation in the application of extended trading hours across the study area. These analyses found that most (63%) premises changed their hours of trade on the implementation date. However, a smaller proportion (15.6%) changed trading times throughout the post-implementation period, potentially delaying the onset of effects. Due to this, the Act’s effect could be abrupt *or* gradual, and therefore it was necessary to estimate each of the transfer functions to determine the most accurate model.

The implementation of the Act, which was introduced at week 96 of the study period (November 24^th^, 2005), was represented as a dummy variable (or *step* function) coded 0 through week 95 and 1 thereafter. As recommended in other studies [37], [51], we also modelled the intervention onset for the 95^th^ and 97^th^ weeks to account for time that might have elapsed before practices were changed. For brevity, these models are only reported where significant effects are detected.

The possibility that the Act had an *abrupt, permanent effect* (1.1) was tested using a zero-order transfer function
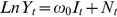
(1.1)


The zero-order transfer function estimates the impact (ω) at the onset of the intervention. Therefore, if the Act had an abrupt one-dimensional effect, the zero-order transfer function would be an adequate model. However, it may be the case that although trading hour restrictions were lifted instantly following the Act’s implementation, intervention effects may not have been immediately observable. Because it is not clear that an abrupt permanent effect should be expected, two additional transfer functions were used to explore alternatives.

The first-order transfer function was used to model each intervention as if the effect of the Act on violence or crime was *gradual, permanent* (1.2):
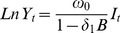
(1.2)


This model estimates the change at onset (ω) as well as a rate value (δ) enabling the identification of dynamic impacts. Using the rate parameter, the asymptotic change (ω/1−δ) can be calculated (i.e. the magnitude of change at the end of the study period). The rate parameter (δ) alone indicates how quickly the overall change was achieved. A value of zero means change was achieved instantly following the onset of an intervention; a value of 1 indicates that the impact was diffused slowly throughout the study period.

As the third alternative for each time series, the Act was modelled as a first-order transfer function applied to a differenced (pulse) intervention variable, thereby testing for the possibility of an *abrupt*, *temporary* (1.3) effect:
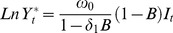
(1.3)


## Results

### General Trends

**Figure 1:**
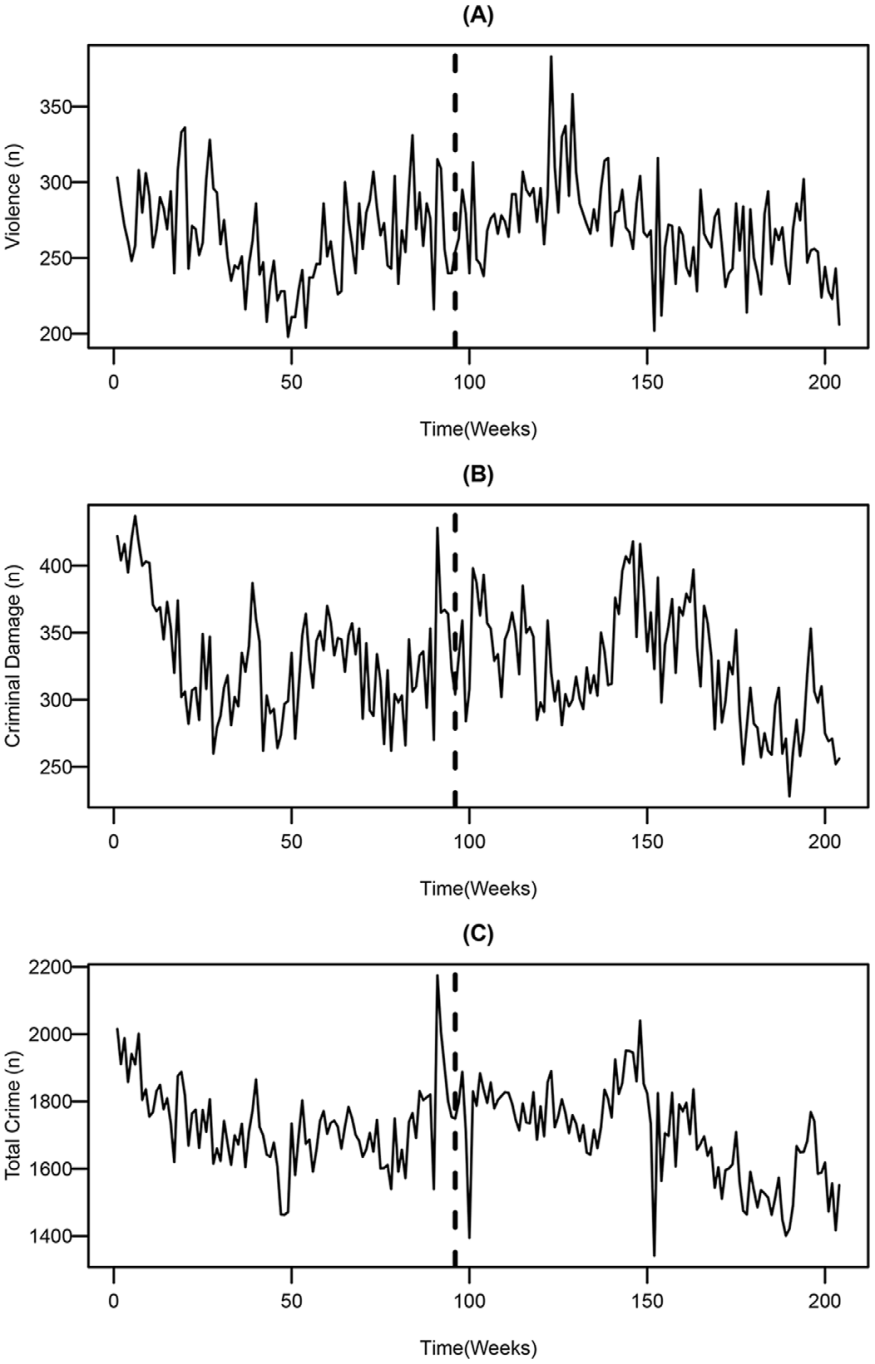
General Crime Trends. Weekly trends in (A) violence, (B) robbery, and (C) total crime, between 2004 and 2008 in the City of Manchester.

Table 1 reports the incidence of violence, robbery, and total crime annually over the four-year study period. The weekly time series of violence shown in Figure 1(a) does not reveal any visual evidence of a distinct disruption to the violence trend following the implementation of the Act in November 2005. When subjected to more rigorous impact assessment, the ARIMA results presented in Table 2 confirm an absence of any significant effects. Estimates using a zero-order transfer function found a non-significant increase of 12% (ω = 0.781, 95% CI = −0.06, 0.17) in violence at the onset of the intervention period (percent changes are calculated using (e^ωz^ *t* –1)100). Furthermore, there was no evidence of a gradual-permanent, or abrupt-temporary effect from models (b) and (c). In these analyses and also in the analyses reported below, parameter estimates derived through maximum likelihood and exact algorithms were indistinguishable.

**Table 2:** ARIMA Interrupted Time Series Parameter Estimates (General Trends).

						95% Confidence Interval		
		ARIMA Model	Parameter	Estimate	SE	Lower	Upper	T-ratio
**Ln Violence**	*a) Zero-Order Transfer Function*	0,1,1	MA (1)	0.781	0.043	0.70	0.86	18.30
	(abrupt-permanent effects)		ω Licensing Act	0.114	0.027	−0.06	0.17	0.43
			ω AMEC	−0.010	0.011	−0.03	0.01	−0.88
			ω Smoking ban	0.010	0.028	−0.05	0.07	0.37
	*b) First-Order Transfer Function (Step)*	0,1,1	MA (1)	0.780	0.046	0.69	0.87	17.03
	(Gradual-permanent effects)		ω Licensing Act	0.007	0.043	−0.08	0.09	0.17
			δ Licensing Act	0.518	0.327	−0.12	1.16	0.16
			ω AMEC	−0.010	0.011	−0.03	0.01	−0.87
			ω Smoking ban	0.010	0.029	−0.05	0.07	0.35
	c) First-Order Transfer Function (Pulse)	0,1,1	MA (1)	0.785	0.045	0.70	0.87	17.36
	(Abrupt-temporary effects)		ω Licensing Act	0.000	0.023	−0.04	0.04	0.02
			δ Licensing Act	0.486	1.635	−2.72	3.69	0.30
			ω AMEC	−0.009	0.011	−0.03	0.01	−0.85
			ω Smoking ban	0.011	0.028	−0.04	0.07	0.38
**Ln Total Crime**	*a) Zero-Order Transfer Function*	1,1,0	AR(1)	−0.450	0.064	−0.58	−0.32	−6.98
			ω Licensing Act	−0.001	0.026	−0.05	0.05	−0.04
	*b) First-Order Transfer Function (Step)*	1,1,0	AR(1)	−0.608	0.066	−0.74	−0.48	−9.27
			ω Licensing Act	0.000	0.014	−0.03	0.03	−0.02
			δ Licensing Act	−0.999	1.609	−4.15	2.16	−0.62
	*c) First-Order Transfer Function (Pulse)*	1,1,0	AR(1)	−0.608	0.066	−0.74	−0.48	−9.27
			ω Licensing Act	0.000	0.014	−0.03	0.03	−0.02
			δ Licensing Act	−0.999	1.609	−4.15	2.16	−0.62
**Ln Robbery**	*a) Zero-Order Transfer Function*	2,1,0	AR(1)	−0.628	0.062	−0.75	−0.51	−10.16
			AR(2)	−0.372	0.075	−0.52	−0.23	−4.98
			ω Licensing Act	−0.084	0.064	−0.21	0.04	−1.32
	*b) First-Order Transfer Function (Step)*	2,1,0	AR(1)	−0.617	0.063	−0.74	−0.49	−9.75
			AR(2)	−0.360	0.075	−0.51	−0.21	−4.77
			ω Licensing Act	−0.037	0.075	−0.18	0.11	−0.05
			δ Licensing Act	−0.956	0.550	−2.03	0.12	−1.74
	*c) First-Order Transfer Function (Pulse)*	2,1,0	AR(1)	−0.611	0.063	−0.73	−0.49	−9.69
			AR(2)	−0.358	0.075	−0.51	−0.21	−4.76
			ω Licensing Act	−0.008	0.024	−0.06	0.04	−0.32
			δ Licensing Act	−0.978	0.111	−1.20	−0.76	−8.81

We extended the search for possible omitted control variables by analysing the impact of the Act on crimes for which it was not expected to have an impact. This is an important validation step as it examines whether some unobserved third variable might have operated on crimes other than violence, hence masking the effect of the intervention. However, Figure 1 (B–C) and Table 3 reveal no evidence of any external shock having an impacted on total crime (−0.09%, ω = 0.001, 95% CI = −0.05, 0.05), or robbery (−8%, ω = −0.084, 95% CI = −0.21, 0.04).

**Table 3:** ARIMA Interrupted Time Series Parameter Estimates (Analysis of Night-Time Patterns of Weekend Violence).

						95% Confidence Interval		
		ARIMA Model	Parameter	Estimate	SE	Lower	Upper	T-ratio
**Ln Violence 18∶00–20∶59**	*a) Zero-Order Transfer Function*	4,1,0	AR(1)	−0.823	0.0467	−0.915	−0.731	−17.61
			AR(2)	−0.6123	0.0703	−0.750	−0.475	−8.70
			AR(4)	−0.3710	0.0846	−0.537	−0.205	−4.38
			ω Licensing Act	−0.1254	0.1039	−0.329	0.078	−1.21
	*b) First-Order Transfer Function (Step)*	4,1,0	AR(1)	−0.8171	0.0500	−0.915	−0.719	−16.35
			AR(2)	−0.6150	0.0729	−0.758	−0.472	−8.43
			AR(4)	−0.3653	0.0883	−0.538	−0.192	−4.14
			ω Licensing Act	−0.2205	0.1457	−0.506	0.065	−1.51
			δ Licensing Act	−0.7179	0.518	−1.733	0.297	−1.39
	c) First-Order Transfer Function (Pulse)	4,1,0	AR(1)	−0.8157	0.0497	−0.913	−0.718	−16.4
			AR(2)	−0.6115	0.0723	−0.753	−0.470	−8.46
			AR(4)	−0.3655	0.0876	−0.537	−0.194	−4.17
			ω Licensing Act	−0.1718	0.1463	−0.459	0.115	−1.17
			δ Licensing Act	−0.4496	0.4994	−1.428	0.529	−0.9
**Ln Violence 21∶00–23.59**	*a) Zero-Order Transfer Function*	0,1,1	MA(1)	0.8834	0.0324	0.820	0.947	27.3
			ω Licensing Act	0.0352	0.0593	−0.081	0.151	0.59
	*b) First-Order Transfer Function (Step)*	0,1,1	MA(1)	0.8779	0.0336	0.812	0.944	26.14
			ω Licensing Act	0.0194	0.0890	−0.155	0.194	0.22
			δ Licensing Act	−0.2375	5.3693	−10.761	10.286	−0.04
	*c) First-Order Transfer Function (Pulse)*	0,1,1	MA(1)	0.8765	0.0334	0.811	0.942	26.24
			ω Licensing Act	0.0044	0.1029	−0.197	0.206	0.04
			δ Licensing Act	−0.8617	10.7467	−21.925	20.202	−0.08
**Ln Violence 00∶00–02∶59**	*a) Zero-Order Transfer Function*	0,1,1	MA(1)	0.8625	0.0342	0.795	0.930	25.24
			ω Licensing Act	0.0369	0.0481	−0.057	0.131	0.77
	*b) First-Order Transfer Function (Step)*	0,1,1	MA(1)	0.8694	0.0324	0.806	0.933	26.84
			ω Licensing Act	0.0404	0.0882	−0.132	0.213	0.46
			δ Licensing Act	−0.1997	5.3163	−10.620	10.220	−0.04
	*c) First-Order Transfer Function (Pulse)*	0,1,1	MA(1)	0.8694	0.0324	0.806	0.933	26.8
			ω Licensing Act	0.0404	0.0882	−0.132	0.213	0.46
			δ Licensing Act	−0.1997	5.3163	−10.620	10.220	−0.04
**Ln Violence 03∶00–05∶59**	*a) Zero-Order Transfer Function*	0,1,1	MA(1)	0.9591	0.0240	0.912	1.006	39.46
			ω Licensing Act	0.0863	0.0623	−0.036	0.208	1.39
	*b) First-Order Transfer Function (Step)* 1	0,1,9	MA(1)	0.8779	0.0336	0.812	0.944	26.14
			MA(2)	−0.2944	0.0174	−0.329	−0.260	56.08
			ω Licensing Act	0.2433	0.0897	0.067	0.419	2.71
			δ Licensing Act	−0.897	0.0634	−1.021	−0.773	−14.14
	*c) First-Order Transfer Function (Pulse)*	0,1,1	MA(1)	0.9276	0.0254	0.878	0.977	36.5
			ω Licensing Act	0.1312	0.1511	−0.165	0.427	0.87
			δ Licensing Act	0.4244	0.6384	−0.827	1.676	0.66

1 Effects shown here were found when the onset of the intervention was lagged 1 week.

Then, to extend the analysis of the time series of violence, we incorporated two policy interventions that may confound the estimation of the Act’s impact: the smoking ban, and a series of AMECs. The smoking ban was entered into the model as a step variable, whereas the AMECs were entered as a series of pulse variables occurring throughout the pre- and post-implementation period. These policy changes occurred close to the change in opening hours. Their omission could therefore bias the estimation of the intervention effects in question. When added to the violence model, neither of these variables were associated with changes to violence (or other crime), and the introduction of these variables had no impact on the model estimates. Similarly, no changes to model estimates occurred when these variables were introduced to the models for total crime, robbery, or criminal damage.

### Impact on Late Night Patterns of Violence

A number of previous studies found evidence of increased violence later in the evening, which could have numerous implications for services that provide support for the night-time economy (i.e. police, emergency services, transport) [22], [26], [27], [31], [33]. In these analyses the aim was to evaluate whether more subtle changes to night-time patterns of violence were observable following the Act’s implementation. This was done by disaggregating night-time patterns of violence into smaller three-hour segments, and conducting ARIMA intervention analysis for each segment.

**Figure 2:**
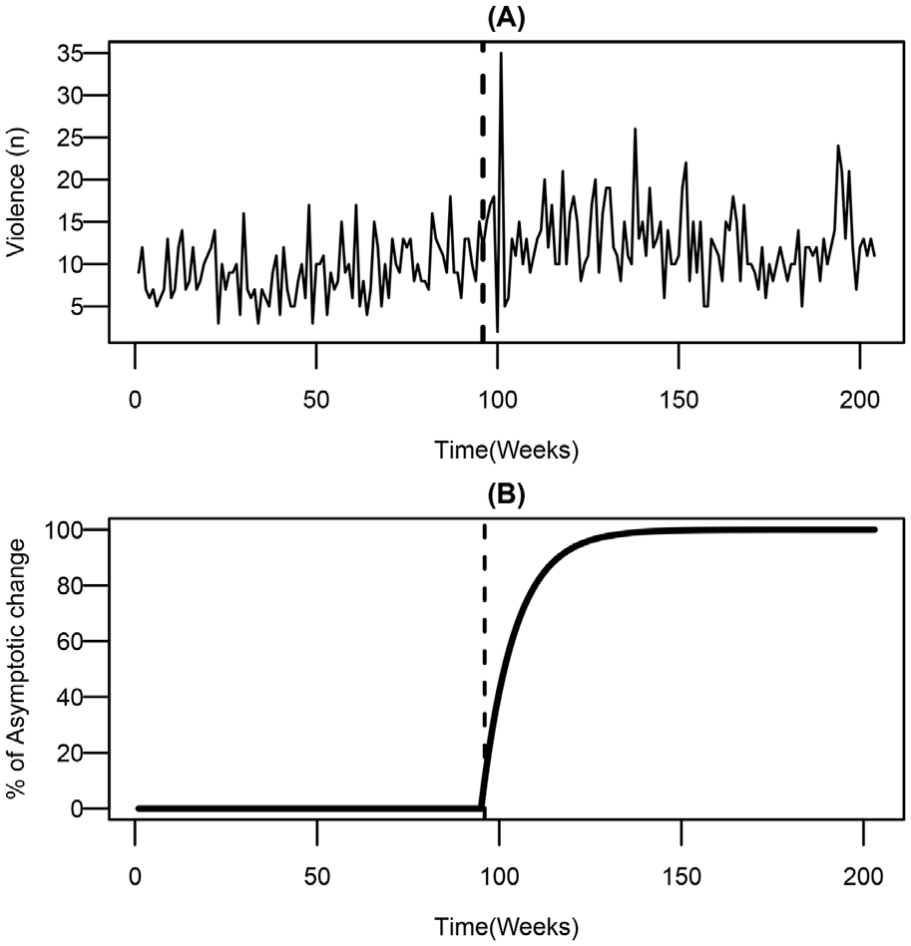
Effect on Weekend Violence (3 a.m. to 6 a.m.). Weekly trends in (A) violence between 3 a.m. and 6 a.m., and (B) the proportion of asymptotic change in violence in the post-intervention period.

The results are presented in Table 3 and Figure 2. For weekdays, ARIMA modelling found no significant changes to violence throughout the evening, and therefore we have not tabulated these results. The findings for weekends are largely supportive of the previous findings and show no statistically significant changes in violence between 6 p.m. and 3 a.m. However, consistent with several other evaluations [26], [27], [31], we found a statistically significant increase in early morning violence between 3 a.m. and 6 a.m.

Specifically, Figure 2(A) shows a series of spikes in the weeks immediately after the Act’s implementation, followed by a subtle increase in the average level of violence. The apparent increase is confirmed in Table 3, where the model for a first-order transfer function shows a significant gradual and permanent impact on violence. The results estimate an initial increase of 27.5% (ω = 0.2433, 95% CI = 0.06, 0.42) at the onset of the intervention. However, the asymptotic change parameter shows that the level of violence continues to increase to a logged rate of 2.36 (asymptotic change = ω/(1−δ))–a 36% increase overall. The significant rate parameter (δ = −0.897, 95% CI = −1.02, −0.77) indicates that the asymptotic level of violence was reached gradually during the post-implementation period. Specifically, this suggests that an increase of 36% overall would predict that the implementation of the Act was ultimately responsible for an additional 3 violent incidents per week between 3 a.m. and 6 a.m. A graph depicting the rate of the impact is presented in Figure 2 (B), showing that the impact was much steeper in the initial weeks following the implementation, but remained significantly higher than pre-implementation levels thereafter.

## Discussion

This study used a natural experimental design to analyse the impact of the removal of trading restrictions on violent behaviour in the City of Manchester. Consistent with other evaluations [26], [27], [28], [29], [39], our findings show no significant changes to the overall level of violence following the implementation of the Licensing Act (2003). Analysis of night-time violence found evidence of a statistically significant 36% increase in violence between 3 a.m. and 6 a.m. These findings add to a growing body of evidence showing that extensions in hourly sales of alcohol may contribute to small yet meaningful changes in late night violence.

These findings also suggested that the Act’s impact was not abrupt, but gradual and permanent. The validity of this finding is strengthened when we consider the findings of a previous study [14] which showed that the Act’s implementation followed a pattern of diffusion similar to that shown in Figure 2(b). Taken together, these findings offer further evidence to reject the government-proposed hypothesis that removing restrictions on trading times would lead to decreased levels of violence. Whilst critics of the Act will argue that the absence of reduced violence is unsurprising in light of the extensive literature associating availability to increased harm [21], [22], it is somewhat surprising that greater *increases* in violence were not observed in this or several other evaluations of the Act (Table S1). Such counterintuitive findings should prompt further investigation of why greater alcohol availability leads to increased violence in some contexts and not in others [52].

Like other studies evaluating the impact of the Licensing Act, this study has a number of strengths and limitations. The ARIMA model approach is arguably a more rigorous method of assessing policy impact than other approaches that have evaluated the Act to date. In this study we explicitly modelled other policy changes that we believe could have had an impact on violence rates during the study period, although it possible that unknown and unmeasured confounding may still exist. We also investigated potential model misspecification by examining whether any change occurred in crime indicators where no impact of the Licensing Act was to be expected. In the absence of overall effects, we also investigated whether more subtle changes to night-time violence were evident. Unlike many previous evaluations, this study was adequately powered to estimate immediate and delayed impacts of the Act.

Power was enhanced in our study by using time series aggregated by week. Relative to monthly time series that we created for comparison to explore this issue, the weekly time series reported above had proportionately better model fits and, hence, supported more powerful hypothesis tests. For example, whereas our weekly time series of late-night violence (3 a.m. to 6 a.m.) suggested that the Act had a gradual permanent impact as evidenced by statistically significant parameter estimates for the transfer function (Table 3), a monthly aggregation of the same data yielded a statistically significant denominator estimate but a numerator estimate that was small in comparison (ω = 0.0475) and included the null value (95% CI = −0.07, 0.12). Also the Q statistic, used to gauge model residuals for randomness at 24 lags, was 18.1 for the monthly time series but 12.0 for the weekly time series. Ultimately then, reliance on monthly data would have led to a different, less defensible conclusion.

The reliance on police-recorded crime data means that these results are susceptible to the ‘dark figure’ of unreported crime [53], [54]. In order to address this, it would have been desirable to supplement the data with injury records from accident and emergency departments, but these data were unavailable. Furthermore, these analyses investigate the impact of extended trading on a macro-level and do not attempt to isolate the relationship between changes to closing times and changes to local rates of violence. As previous studies have shown [55], the implementation of the extended trading hours differed across cities, meaning that smaller geographic units are likely to have experienced different doses of the intervention. It is conceivable that violence increased (or decreased) in areas where extended trading increased, and reduced in areas where trading contracted, thus averaging out effects when examined at the macro level.

A further limitation of this study is its generalisability. Under ideal circumstances, it would have been desirable to design a prospective multisite study, collecting detailed population data on alcohol consumption patterns, licensed trading times and both self-reported and recorded rates of violence and injury. However, given the nature of the Act’s implementation [14], the considerable resources this would require, and the lack of detailed routine data, such a design was deemed infeasible. Complex policy interventions like this are rarely able to deal with all threats to external validity [36], and in many cases, generalisable causal inference will rely on the replication of natural experiments and the synthesis of this combined evidence [56]. By increasing attention to the rigor and internal validity of this evaluation, this study provides further evidence that the Act failed to reduce violence (as was suggested by the Labour government), and may have contributed to additional problems by spreading violence later into the early hours of the morning.

The findings from this and other evaluations of the Act have implications both for policymakers and the research community. These findings serve as a reminder that preventive policy may not always have its anticipated affect on behaviour, and when conceived without proper attention to available scientific evidence, may even cause harmful side effects. These findings suggest some evidence of negative side effects on early morning violence resulting from the Act’s implementation. However, we still know very little about whether an absence of dramatic effects on overall violence is evident in other alcohol-related harms. Future research needs to move beyond black-box evaluative designs by investigating the impact of policy on exposure (e.g. alcohol availability) as well as the impact of exposure on multiple indicators of physical and social harm. In England and Wales, a vast improvement in the provision of routine data on alcohol availability and consumption is required to further our understanding of the relationship between alcohol availability, alcohol misuse and its related harms.

## Supporting Information

Table S1 **Summary of Evaluations of Extended Trading.** This table provides a summary of the findings of 13 evaluations of interventions extending the availability of alcohol.
(DOC)
