# Supplementary material for: Evaluating the Impact of Flexible Alcohol Trading Hours on Violence: An Interrupted Time Series Analysis
Source: PLoS One. 2013 Feb 15;8(2):e55581. doi: 10.1371/journal.pone.0055581 (PMC3574094; doi:10.1371/journal.pone.0055581)
Supplement: Table S1 — Summary of Evaluations of Extended Trading. This table provides a summary of the findings of 13 evaluations of interventions extending the availability of alcohol. (DOC) [file pone.0055581.s001.doc]

| **Supplementary Table S1: Summary of Evaluations of Extensions to Trading Hours** | | | | | | | |
| --- | --- | --- | --- | --- | --- | --- | --- |
| **Study** | **Study Design** | **Unit(s) of Analysis** | **Data Source** | **Outcome Measures** | **Main Findings** | **Peer reviewed** | **Directional change in violence** |
| Duffy et al., (1996) | Before-after, control region. | Regional, England and Wales | Police: recorded crime data | Violent crime | Non-significant increase in recorded violent crime 15.5% (95% CI:14.0%, 17.0%). | Yes | ↔ |
| Ragnardottir et al., (2002) | Before-after, no comparison | City centre, Reykjavik, Iceland. | Emergency department attendances | Weekend evening attendances | Change in total attendances (+3%), change on evaluation nights (+31%), change on weekends (+20%), change on weekdays (-2%). Significance tests not reported. | No | ↑ |
| Chikritzhs et al., (2002) | Interrupted time series design | 188 Hotels in Perth, Aus. | Police: recorded crime data | Violent assault, Alcohol consumption. | Significant(p<.01) increase in violent assault in the treatment area (54.5%) versus comparison areas (18.7%).Coincided with measured increases in purchase of high-strength alcohol. | Yes | ↑ |
| *Bellis et al., (2006) | Before and after, no comparison. | Regional Unit: Wirral | Emergency department: A&E admissions | Violent assault | Significant (p<.001) decrease in violent assault compared to a pooled before period. Significant decrease in violent assault compared to the previous year. | No | ↓ |
| *Babb (2007) | Before and after, no comparison. | a) National Unit: Pooled results for 30 police forces, b) Subset of City Centre Units: 18 Police Forces | Police: recorded crime data | More serious violence, less serious wounding, less serious wounding in city centres (subset), assault with no injury (pooled & subset), harassment (pooled & subset), criminal damage | No significance tests: 22% increase between 3 a.m-6 a.m.; 'Less serious wounding' = -5% overall decrease, and 26% increase between 3 a.m.-6 a.m. (for subset of city centres this was a 133% increase); 'Assault with no injury' = -2% overall decrease, and a 22% increase between 3 a.m.-6 a.m. (for subset of city centres this was a 123% increase). Significance tests not reported. | No | ↔ |
| *Newton et al., (2007) | Before and after, no comparison. | City Centre London: Undefined catchment area for St. Thomas' hospital | Emergency department: alcohol-related Admissions | Alcohol related attendances, Alcohol assault and injury attendances | Significant increases in 'Total number of alcohol-attendances' (5.1%); 'Alcohol related assault' (1%); 'Alcohol related injury' (2.5%); 'Alcohol related hospital admission' (1.58). | Yes | ↑ |
| *Durnford et al., (2008) | Before and after, no comparison. | City Centre Birmingham: Undefined catchment area for Birmingham emergency department | Emergency department: alcohol-related admissions | Total weekly attendances | No significant change in the volume of violent assault.. Significant change in the temporal distribution of weekly assault= 44% increase in weekend offending; and a 27.3% increase in offending between 3a.m.-9a.m. | Yes | ↔ |
| *Newton et al.,(2008)/ Hough & Hunter., (2008) | Before and after, multi-site study | Multiple units: Macro (City), Meso (Cluster), Micro (Individual bar) | Police: recorded crime data, emergency department: A&E data, qualitative research | Police: violence against the person; criminal damage, disorder calls for service. A&E: violent admissions. | Violence Against the Person'= Significant change in 1 out of 5 study sites (Nottingham=2.8%, p<.001) | Yes | ↔ |
| *Pike et al., (2008) | Before and after, multi-site study | 1 City and 2 Town Centre Units | Police: recorded crime data. | Take up and use of extended hours, Changes in workloads and practices, Change in drinking behaviour, Change in crime and disorder. Change in the time of offence. | No significant change in crime and disorder (reanalysed = Mean difference 1.5, t=.95, p= n.s.). | No | ↔ |
| *El-Maaytah et al., (2008) | Before and after, no comparison. | City Centre London: Undefined catchment area for University College Hospital (UCH) | Emergency department: alcohol-related trauma admissions | Head and neck trauma presentations at A&E. | Significant 34% reduction in A&E cases of alcohol-related head and neck trauma following the Act's implementation. | Yes | ↓ |
| *Jones & Goodacre (2010) | Before and after, multi-site study | Undefined catchment areas for 4 Emergency departments in South Yorkshire | Emergency department: attendances | Alcohol related attendances (clinical coding) | Significant increase in 'alcohol-related attendances' of 0.1% (95% CI 0.1-0.2, p<.0001). | Yes | ↑ |
| *Pierce & Boyle (2011) | Before and after, no comparison. | South Cambridgeshire: Undefined catchment area for Cambridge emergency department | Emergency department attendances | Assault attendances (Before/ After), domestic violence, change in time of assault attendances. | Significant increase of 12.3% (z=1.95, p=0.05) total assaults; Significant decrease (χ^2^=16.82, df=1, p<0.001) in the proportion of women assaulted; slight increase in presentations at weekends (χ^2^=35.95, df=6, p<0.001); significant increase in assault presentation (Two-sample Wilcoxon rank-sum test, p=0.004) after midnight and before 8 a.m. | Yes | ↑ |
| *Kirby & Hewitt (2011) | Before and after, no comparison. | Preston, England | Police: recorded alcohol-related crime. | Alcohol-related violence | An average decrease of 33% in alcohol related crime in the post-intervention period. A 55% increase in the average number of alcohol related offences occurring between 3 a.m. to 4 a.m. Significance tests not reported. | Yes | ↓ |
| Rossow & Norstrom (2012) | Interrupted time series design, inner city areas (treatment), outer city areas (control) | 18 Norwegian cities | Police: recorded crime data | Violent assault | Statistically significant increase of 5.0 assaults per 100,000 per quarter (17%, 95% CI: 11% - 24%). | Yes | ↑ |
| * denotes studies evaluating the impact of the Licensing Act (2003) | | | | | | | |
